# Metabolic Reprogramming of Macrophages by Walnut Peptides: Interference with Arginine-to-Creatine Conversion

**DOI:** 10.3390/foods15214014

**Published:** 2026-11-09

**Authors:** Yang Chen, Peng Deng, Xing Shen, Shuang Zhang, Jingxuan Guo, Dan Li, Zhiyong He, Jie Chen, Maomao Zeng

**Affiliations:** 1 State Key Laboratory of Food Science and Resources, Jiangnan University, Wuxi 214122, China; c756542878@163.com; 2 School of Food Science and Technology, Jiangnan University, Wuxi 214122, Chinazyhe@jiangnan.edu.cn (Z.H.); chenjie@jiangnan.edu.cn (J.C.); 3 College of Resources and Environment, Xinjiang Agricultural University, Urumqi 830052, China; shenxing@xjau.edu.cn; 4 Analysis and Testing Center, Jiangnan University, Wuxi 214122, China

**Keywords:** walnut, peptides, arginine metabolism, macrophage, guanidinoacetic acid

## Abstract

Walnut protein is a promising candidate for the development of functional health supplements. In this study, peptides were prepared via the limited trypsin hydrolysis of walnut protein, and their immunomodulatory effects on macrophages were evaluated through both in vivo and in vitro experiments. We found that peptide administration modulated systemic arginine metabolism. In macrophages, the peptides induced transcriptional reprogramming toward a pro-inflammatory phenotype, regardless of their immunosuppressive status. Specifically, peptide treatment upregulated *Slc7a2* expression, concomitant with enhanced arginine uptake, while concurrently suppressing the arginine-to-guanidinoacetic acid/creatine pathway. These findings reveal a mechanistic role of walnut-derived peptides in regulating macrophage polarization through arginine metabolic reprogramming. Consequently, the stimulation of M2-polarized macrophages with these peptides reversed their tumor-promoting propensity and restored their cytotoxicity against tumor cells. Together, these findings provide a scientific basis for the value-added processing and high-end utilization of walnut proteins in nutraceutical applications.

## 1. Introduction

The nut industry constitutes a substantial segment of the global agricultural economy. Among the various types of nuts, walnut, hazelnut, almond, and cashew are collectively referred to as the “four major nuts” and have undergone a substantial increase in cultivation scale in recent years due to their high nutritional and economic value. This growth has, in turn, driven the demand for transformation and upgrading within the nut industry. In earlier stages, the primary utilization pathway for nuts was direct consumption, followed by processing into healthy oils [1]. This phenomenon is attributed to their higher lipid content compared to traditional bulk oil crops [2] and the demonstrated health-promoting effects of their oils [1,3]. As a byproduct of oil processing, nut meals are currently commercialized in limited forms, primarily for use in animal feed and plant fertilizer applications. Protein constitutes the primary component of nut meals. In the context of population growth accompanied by protein resource shortages and the increasingly prominent challenge of an “increase in production without a corresponding rise in income” in the nut industry, the high-value utilization and development of nut protein have become a focal point in food research. China is the world’s leading producer of walnuts, and significant research and development has been dedicated to the deep processing of walnut meal. A substantial body of research has been dedicated to investigating the processing characteristics of walnut protein and its potential as a substitute for alternative protein sources in existing food systems [4]. These efforts have effectively promoted the application of walnut protein in the food sector and enhanced consumer acceptance. However, foods developed solely as substitutes for existing proteins lack the capacity to enhance the high value-added potential of walnut protein. In the future, research will continue to prioritize the study of dietary behavior that promotes human health and longevity, with a focus on outcomes beyond basic nutritional needs. Consequently, research in this direction could substantially enhance the value-added potential of walnut protein.

Previous studies have reported that walnuts possess immunomodulatory properties, which are closely associated with their lipid [5], polyphenolic flavonoid [6], and protein components [7]. Peptides, as protein-derived moieties, possess molecular weights intermediate between those of intact macromolecular proteins and small phenolic compounds and exhibit diverse physicochemical and biological characteristics. Moreover, following appropriate modification, these peptides can be endowed with potent targeting specificity [8], often conferring advantages over other functional constituents. This provides substantial opportunities for enhancing the added value of walnut proteins through advanced processing technologies. Although the immunomodulatory effects of walnut-derived peptides have been documented, current research has predominantly focused on their anti-inflammatory activities. This focus stems largely from the prevailing notion that mitigating inflammation is beneficial for managing diseases arising from chronic inflammation [9,10]. Peptides prepared using various proteases including trypsin, acid protease, alkaline protease, and other proteases have consistently been shown to inhibit the secretion of inflammatory cytokines from immune cells [11]. These findings robustly confirm the presence of immunosuppressive core sequences within walnut proteins, which can be released via multiple enzymatic treatments. Notably, however, tryptic hydrolysates of walnut protein have also been reported to promote the secretion of inflammatory cytokines [12], thereby exhibiting immunostimulatory properties. This evidence indicates the existence of immune-enhancing amino acid motifs within the same protein source. Such dual (i.e., both anti-inflammatory and immunostimulatory) effects of proteolytic peptides have been corroborated in other studies [13,14,15]. Consequently, future processing strategies for walnut peptides should aim to selectively preserve specific amino acid sequences tailored for either anti-inflammatory or immunostimulatory purposes. This selective approach is critical to avoid the regulatory and applicative uncertainties associated with overly complex heterogeneous peptides mixtures, which could otherwise limit their targeted applicability in immunomodulation.

Robust and sustained activation of the immune system is urgently required in the context of diseases such as cancer [16], acquired immunodeficiency syndrome [17], and syphilis [18]. Among these conditions, the interplay between tumors and the host immune system is particularly complex. During early oncogenesis, the immune system effectively surveils and eliminates neoplastic cells. However, through continuous adaptive remodeling, tumor cells successfully subvert host immune defenses, rendering the immune response tumor-promoting in advanced stages of progression and metastasis—a phenomenon closely associated with the extensive infiltration of immunosuppressive cells [19]. Macrophages, as frontline sentinels of both innate and adaptive immunity, play a pivotal role in this process. Mechanistic studies on tumor progression and metastasis have identified macrophages as one of the earliest immune cell populations influenced by tumors [20], which subsequently undergo polarization toward an immunosuppressive phenotype. These polarized macrophages further accelerate tumor malignancy by recruiting additional immunosuppressive cell subsets and suppressing the cytotoxic function of tumor-killing CD8^+^ T cells [21]. Although earlier studies have demonstrated that walnuts [22] and their derived peptides [23,24] can inhibit tumor cell proliferation, the direct cytotoxic efficacy of walnut peptides varies considerably across different cancer types, owing to substantial inter-tumor heterogeneity. Moreover, tumor heterogeneity and the emergence of adaptive resistance present major limitations to the development of walnut peptides as specialized medical foods for cancer patients. Therefore, harnessing walnut-derived peptides to enhance immune surveillance and tumor clearance represents a promising strategy to effectively circumvent these constraints.

It should be noted that the immunostimulatory peptides validated in previous studies were predominantly evaluated using cyclophosphamide (CTX)-induced immunocompromised mouse models [25,26] or unpolarized macrophages in vitro [27], which fundamentally differ from the immunostimulatory requirements pertinent to cancer patients. The CTX model is primarily characterized by a profound depletion of immune cell populations, resulting in systemic immune incompetence. Similarly, screening protocols based on unpolarized macrophages primarily assess the capacity of peptides to promote macrophage proliferation and induce the release of inflammatory cytokines—outcomes that essentially reflect a quantitative restoration of innate immune responsiveness rather than a qualitative functional correction [7,25,26]. In stark contrast, the immunosuppressive state within the tumor microenvironment (TME) arises from the cytokine-driven metabolic and functional reprogramming of infiltrating immune cells, leading to a deviation from their expected anti-tumorigenic phenotypes. This immunosuppression is not attributable to a numerical deficiency of immune cells, but rather to an acquired dysfunction characterized by aberrant metabolic pathways and impaired effector functions. Consequently, peptides previously reported to possess general immunostimulatory properties are not necessarily applicable to the reversal of immunosuppressive phenotypes to restrain tumor progression. This distinction underscores that strategies aimed at simply boosting immune cell numbers or baseline activity may be insufficient and that the development of peptide-based interventions for oncology applications must specifically target the reprogramming of functionally deviated immune cells within the TME. This study investigates the effects of walnut-derived peptides on reprogramming immunosuppressive macrophages toward an inflammatory (M1-like) phenotype and elucidates the underlying mechanisms, thereby addressing a critical gap in the application of food-derived bioactive peptides for tumor-immunology-oriented special medical foods. Our findings will provide a theoretical foundation for the development and utilization of high-value-added specialized medical foods derived from walnut proteins.

## 2. Materials and Methods

### 2.1. Materials

Walnuts, farmed in Xinjiang, were obtained from Taobao (https://www.taobao.com). Trypsin, from porcine pancreas, 2500 units/mg, was purchased from J&K Scientific Co., Ltd. (Shanghai, China). The RAW264.7, CT26, LLC, 4T1-LUC, and MC38 cell lines were sourced from Shanghai Zhong Qiao Xin Zhou Biotechnology Co., Ltd. (Shanghai, China). The immortalized bone marrow-derived macrophage (iBMDM) and Hep 3B2.1-7 cell lines were obtained from Guangzhou Editgene Technology Co., Ltd. (Guangzhou, China). The Hep G2, PC12, and SCC7 cell lines were purchased from Haixing Biosciences (Guangzhou, China). Cell culture media were bought from Shanghai Zhong Qiao Xin Zhou Biotechnology Co., Ltd. (Shanghai, China).

### 2.2. The Preparation of Peptides

According to the method of Mao and Hua [28], walnuts were defatted using n-Hexane at a ratio of 1:5 (*w*/*v*) for 2 h per treatment, repeated three times. The defatted walnut powder was mixed with 26 volumes of deionized water, the pH was adjusted to 11 using 4 mol/L NaOH, and the mixture was stirred at room temperature for a period of 4 h. Following centrifugation at 8000× *g* for 20 min, the supernatant was collected and the pH was adjusted to 4.5 using 4 mol/L HCl. The precipitate was collected by means of centrifugation, redissolved in water while adjusting the pH to 8, and subsequently freeze-dried. The resultant product was the walnut protein isolate.

A 50 mg/mL walnut protein isolate was prepared for subsequent enzymatic hydrolysis. The protein solutions were pre-incubated at 37 °C, followed by the addition of trypsin to achieve 0, 0.01, 0.05, 0.1, 0.5, and 1 mg/mL. After enzymatic hydrolysis for 5 min, the mixture was boiled for 15 min to terminate the reaction. Following centrifugation at 8000× *g* for 20 min, the supernatant was subjected to ultrafiltration using a 30 kDa membrane. The filtrate was collected and freeze-dried. The resulting product was stored at −80 °C until further use.

### 2.3. Protein Content of Walnut Protein Isolate and Peptides

The protein content of the sample was determined using the micro-Kjeldahl method carried out on a K9840 automatic Kjeldahl nitrogen analyzer (Hanon Advanced Technology Group Co., Ltd., Jinan, China), using a protein-to-nitrogen conversion factor of 5.30.

### 2.4. Quantitation of Amino Acid Profile

According to the method of Fang and Chen [29], a 100 mg sample was weighed into an acid hydrolysis tube, with 8 mL of 6 mol/L HCl added. Following 3 min of N2 purging, the sealed tube was subjected to hydrolysis at 140 °C for 4.5 h. The sample was neutralized with 4.8 mL of 10 mol/L NaOH and diluted to 50 mL. The resultant solution was filtered through a 0.22 μm nylon membrane before chromatographic analysis.

The amino acids were quantitated on an Agilent 1100 Series HPLC with a G1314A variable wavelength detector, using a Agilent Hypersil ODS (4.0 × 250 mm, 5 μm) column at 40 °C and flow rate of 1.0 mL/min with Solvent A (27.6 mmol/L sodium acetate/triethylamine/tetrahydrofuran = 500/0.11/2.5, *v*/*v*/*v*, pH = 7.2) and Solvent B (80.9 mmol/L sodium acetate/methanol/acetonitrile = 1/2/2, *v*/*v*/*v*, pH = 7.2).

### 2.5. SDS-PAGE

The molecular weights of the walnut protein isolate and peptide bands were analyzed using SDS-PAGE (GSG2001-420T, Shanghai Wansheng Haotian Biotechnology Co., Ltd., Shanghai, China) and Tricine-SDS-PAGE according to the method of Schägger [30]. The high-molecular-weight (MW) standard protein marker (MKPX011, Shanghai Wansheng Haotian Biotechnology Co., Ltd., Shanghai, China) and low-molecular-weight standard protein marker (MKPX012, Shanghai Wansheng Haotian Biotechnology Co., Ltd., Shanghai, China) were used to as standard bands. Proteins or peptides were visualized by staining the gel with a rapid Coomassie Brilliant Blue staining solution (EZWB19, Shanghai Wansheng Haotian Biotechnology Co., Ltd., Shanghai, China).

### 2.6. Size-Exclusion High-Performance Liquid Chromatography (SEC-HPLC)

The SEC-HPLC was carried on a Waters e2695 separation module coupled with a Waters 2998 diode array detector at the wavelength was set at 210 nm. The MW analysis of walnut protein isolate was performed on a Shodex PROTEIN KW-804 (8.0 mm I.D. × 300 mm) with 1 mL/min of solvent (50 mM Sodium phosphate buffer (pH7.0) + 0.3 M NaCl). The MW analysis of peptides was performed on a TSKgel^®^ G2000SWXL (7.8 mm I.D. × 300 mm, 5 µm) with 0.5 mL/min solvent (acetonitrile/H_2_O/trifluoroacetic acid = 45/55/0.1). Standard materials included the following: Gly-Gly-Gly, MW—189 Da; Gly-Gly-Tyr-Arg, MW—451 Da; Bacitracin, MW—1422 Da; Aprotinin Hydrochloride, MW—6500 Da; Cytochrome C, MW—12,327 Da; Myoglobin, MW—17,000 Da; Albumin, MW—67,000 Da; Thyroglobulin, MW—669,000 Da.

### 2.7. Peptide Desalting and LC-MS/MS-Based Identification

The peptides were desalted using a peptide desalting spin column (89851, Thermo Fisher, Rockford, IL, USA). The peptide concentration was then adjusted to 1 μg/μL, and 0.4 μL was used for mass spectrometric identification. Chromatographic separation was performed using a Vanquish Neo UHPLC system (Thermo Fisher Scientific, San Jose, CA, USA). Mobile phase A was 0.1% formic acid in water, and mobile phase B was 0.1% formic acid in 80% acetonitrile. The analytical column was equilibrated with 96% mobile phase A. Samples were loaded onto a trap column (PepMap Neo 5 µm C18, 300 µm × 5 mm; Thermo Scientific) and then separated on an analytical column (μPAC Neo High Throughput column; Thermo Scientific) using the following gradient: 0–0.1 min, 4–8% B; 0.1–1.2 min, 8–10% B; 1.2–11.2 min, 10–28% B; 11.2–13.5 min, 28–45% B; and 13.5–15 min, 99% B. The separated peptides were analyzed via data-dependent acquisition (DDA) on an Orbitrap Astral mass spectrometer (Thermo Scientific). The total acquisition time was 15 min. The electrospray voltage was 2.2 kV, and positive ion mode was used. The precursor scan range was 380–980 m/z. The MS1 resolution was 240,000, with an AGC target of 500% and a maximum injection time of 3 ms. The MS2 resolution was 80,000, with an AGC target of 500% and a maximum injection time of 3 ms. The RF lens was set to 40%. Higher-energy collisional dissociation (HCD) was used for MS2 activation, with an isolation window of 2 Th, a normalized collision energy of 25%, and a cycle time of 0.6 s. Raw data were processed using the de novo peptide sequencing module of PEAKS Studio 11.5 (Bioinformatics Solutions Inc., Waterloo, Canada). Peptides with blank peak areas in the identification results were removed, and sequences with an average local confidence (ALC) ≥90% were considered high-confidence peptides.

### 2.8. Determination of PTIO•-Scavenging Activity

According to the method of Li [31] and Chen and Xi [32], the peptides was mixed with 3 mL of a 40 μg/mL PTIO• (P838429, Shanghai Macklin Biochemical Technology Co., Ltd., Shanghai, China) solution at 37 °C for 24 h. Absorbance of the reaction mixture was measured at 557 nm with a SpectraMax 190 Microplate Reader (Molecular Devices, LLC, San Jose, CA, USA).

### 2.9. Cell Viability Assay

The effect of the peptides on the cell lines was assessed using a CCK-8 kit (MeilunBio, Dalian, China). Cells were seeded at 1 × 10^4^ cells/well and incubated at 37 °C for 24 h. Subsequently, the cells were incubated with serially diluted peptide solutions for 24 h. After washing twice with PBS, the cells were incubated with CCK-8 reagent for 2 h, and the absorbance was measured at 450 nm with a reference wavelength of 630 nm. Cell viability was determined according to the manufacturer’s instructions. Preliminary experiments showed that the maximum tolerated concentrations of the peptide were 1 mg/mL for iBMDM cells and 2 mg/mL for RAW264.7 cells. Unless otherwise stated, all subsequent cell experiments were performed with a peptide concentration of 1 mg/mL.

### 2.10. Cell Models

According to the methods of Guo and Ji [33] and Lundahl and Mitermite [34], RAW264.7 and iBMDM cells were treated with 100 ng/mL lipopolysaccharide (LPS) (biosharp, Beijing, China) for 24 h to induce M1 macrophage polarization. To induce M2 polarization, macrophages were treated with 20 ng/mL IL4/IL13 (90144ES50 and 90248ES50, Yeasen Biotechnology (Shanghai) Co., Ltd., Shanghai, China) for 48 h.

### 2.11. Macrophage and Tumor Cell Co-Culture Models

According to the method of Zhang and Xie [35], iBMDM cells were indirectly co-cultured with MC38 cells using Transwell^®^ plates with a pore size of 0.4 μm (TCS001012, Guangzhou Jet Bio-Filtration Co., Ltd., Guangzhou, China). For M2 polarization, iBMDM cells (1 × 10^5^) treated with 1 mg/mL peptide for 24 h and then rinsed with PBS were grown in the lower chamber, while MC38 cells (5 × 10^4^) were grown in the upper chamber and co-cultured for 24 h.

### 2.12. Animal Experiments

The experimental procedures of this study were conducted in strict accordance with the guidelines for the ethics and welfare of laboratory animals of Jiangnan University, and specific pathogen-free (SPF)-grade 6-week-old male C57BL/6J mice were provided by Beijing Vital River Laboratory Animal Technology Co., Ltd., Beijing, China. All experimental protocols were approved by the Institutional Animal Ethics and Welfare Committee of Jiangnan University (License JN. No 20250228m1140424[022]). The mice were maintained in a controlled environment with a temperature of 23 ± 3 °C, a 12 h light/12 h dark cycle, and relative humidity of 60 ± 5%. After a one-week acclimatization, the mice were randomly divided into two groups (n = 12): a normal diet group and a peptide diet group (1.7 g/kg peptide). The peptide group received the peptide via daily gavage, whereas the normal diet group received PBS via daily gavage. Gavage was performed for 2 consecutive weeks. Mice were then euthanized for tissue collection.

### 2.13. RNA Extraction and RT–qPCR

The cell and tissue RNA were extracted using a total RNA isolation reagent (SM130, biosharp, Beijing, China). cDNA synthesis was performed using a Hifair^®^ III 1st Strand cDNA Synthesis SuperMix for qPCR (gDNA digester plus) (Yeasen Biotechnology (Shanghai) Co., Ltd, China). RT–qPCR was performed using the Hieff UNICON^®^ ColorGPS qPCR SYBR Green Master Mix (No Rox) (Yeasen Biotechnology (Shanghai) Co., Ltd, Shanghai, China). All primer sequences utilized in RT–qPCR are shown in Supplementary Table S1.

### 2.14. Untargeted Metabolomics Analysis

Fifty milligrams of animal tissue was weighed and mixed with 400 μL of pre-cold methanol. Subsequently, the samples were homogenized on a high-throughput tissue homogenizer (SCIENTZ-48, Ningbo Scientz Biotechnology Co., Ltd., Ningbo, China) at 50 Hz and 10 s, 4 times. After centrifugation at 12,000× *g* for 20 min, the supernatants were transferred into a new 1.5 mL tube for concentration and drying, carried out on a CentriVap vacuum centrifugal concentrator (Labconco Corporation, Kansas City, MO, USA). Prior to instrumental analysis, the lyophilized samples were reconstituted with 100 μL of a 20% methanol aqueous solution. Equal volumes of supernatant from each group of samples to be analyzed were collected, pooled, and vortex-mixed to prepare quality control samples. The pseudotargeted metabolomics analysis was carried according to our previous method [36]. The untargeted metabolomics analysis was carried on an Orbitrap Exploris™ 240 (Thermo Scientific, USA) coupled with Thermo Hypersil Gold (1.9 μm, 2.1 × 100 mm); column temperature was 50 °C. The mobile phase was acetonitrile in phase A and water (containing 0.1% formic acid) in phase B. The flow rate of the mobile phase was 0.3 mL/min, and the injection volume was 2 μL. The chromatographic gradient elution program was as follows: 0 min, 95% B; 1 min, 95% B; 13 min, 1% B; 15 min, 1% B; 15.1 min, 95% B; 18 min, 95% B. The mass spectrometry parameters of positive ion scan mode settings were as follows: mass scan range (m/z), 70–1050 Da; ion source, ESI; spray voltage, 3.5 kV; sheath gas flow rate, 40 arb; aux gas flow rate, 10 arb; capillary temperature, 275 °C; and the data acquisition mode of mass spectrometry was data-dependent acquisition (DDA) mode. The mass spectrometry parameters of negative ion scan mode settings were as follows: mass scan range (m/z), 70–1050 Da; ion source, ESI; spray voltage, 2.8 kV; sheath gas flow rate, 40 arb; aux gas flow rate, 10 arb; capillary temperature, 275 °C; and the data acquisition mode of mass spectrometry was DDA mode.

### 2.15. Targeted Metabolomics Analysis

The method was adapted from Guo and Li [37] with modifications. Briefly, 50 μL of the supernatants was mixed with 30 μL of an ethylenediamine–sulfonyl chloride acetonitrile solution (10 mg/mL) and 40 μL of a 0.5 M sodium carbonate–sodium bicarbonate buffer. The mixture was incubated in a 60 °C water bath for 30 min. Then, 10 μL of 0.25 M NaOH was added, and the mixture was reacted at 60 °C for 10 min. After cooling to room temperature, 70 μL of a 10% formic acid solution was added, and 1 μL of the mixture was injected for analysis.

Analysis was performed on an MS8000 triple quadrupole tandem mass spectrometer (GAMSTEK Co., Ltd., Hefei, China) coupled with a Phenomenex Kinetex C18 column (2.6 μm, 2.1 × 50 mm) at a column temperature of 40 °C. The mobile phase consisted of acetonitrile containing 0.1% formic acid (phase A) and water containing 0.1% formic acid (phase B). The flow rate was 0.17 mL/min. The gradient elution program was as follows: 0 min, 85% B; 3.5 min, 65% B; 6 min, 60% B; 8.5 min, 58% B; 10.5 min, 40% B; 11.5 min, 5% B; 12 min, 5% B; 12.5 min, 0% B; 13 min, 0% B; 13.1 min, 85% B; 15 min, 85% B.

### 2.16. Transcriptomic Analysis

The Trizol method (15596018, Thermo fisher, Waltham, MA, USA) was employed to isolate total RNA. The purified RNA was then synthesized into cDNA using SuperScript II Reverse Transcriptase (Invitrogen, cat. 1896649, Carlsbad, CA, USA) and E. coli DNA polymerase I (NEB, cat.m0209, Ipswich, MA, USA). To prepare the fragments for sequencing analysis, dual-index adapters were ligated followed by PCR amplification. Finally, the cDNA libraries were sequenced on an Illumina Novaseq™ 6000 platform (LC-Bio Technology CO., Ltd., Hangzhou, China). For bioinformatics analysis, a cDNA library was created by combining RNA samples from various sources of research species and subjected to sequencing on an Illumina Novaseq™ 6000 platform. The obtained reads underwent filtration using Cutadapt (https://cutadapt.readthedocs.io/en/stable/, version: cutadapt-1.9 (access on 8 July 2025)), followed by alignment to the reference genome utilizing HISAT2 (https://daehwankimlab.github.io/hisat2/, version: hisat2-2.2.1 (accessed on 8 July 2025)).

### 2.17. Statistical Analysis

All summary data are expressed as the mean ± standard deviation. Mann–Whitney U tests were performed on independent samples to determine the significance of differences between samples by using SPSS 29 software (SPSS Inc., Chicago, IL, USA). Differences of *p* < 0.05 were statistically significant.

## 3. Results

### 3.1. Chemical Structure of Peptides

**Figure 1:**
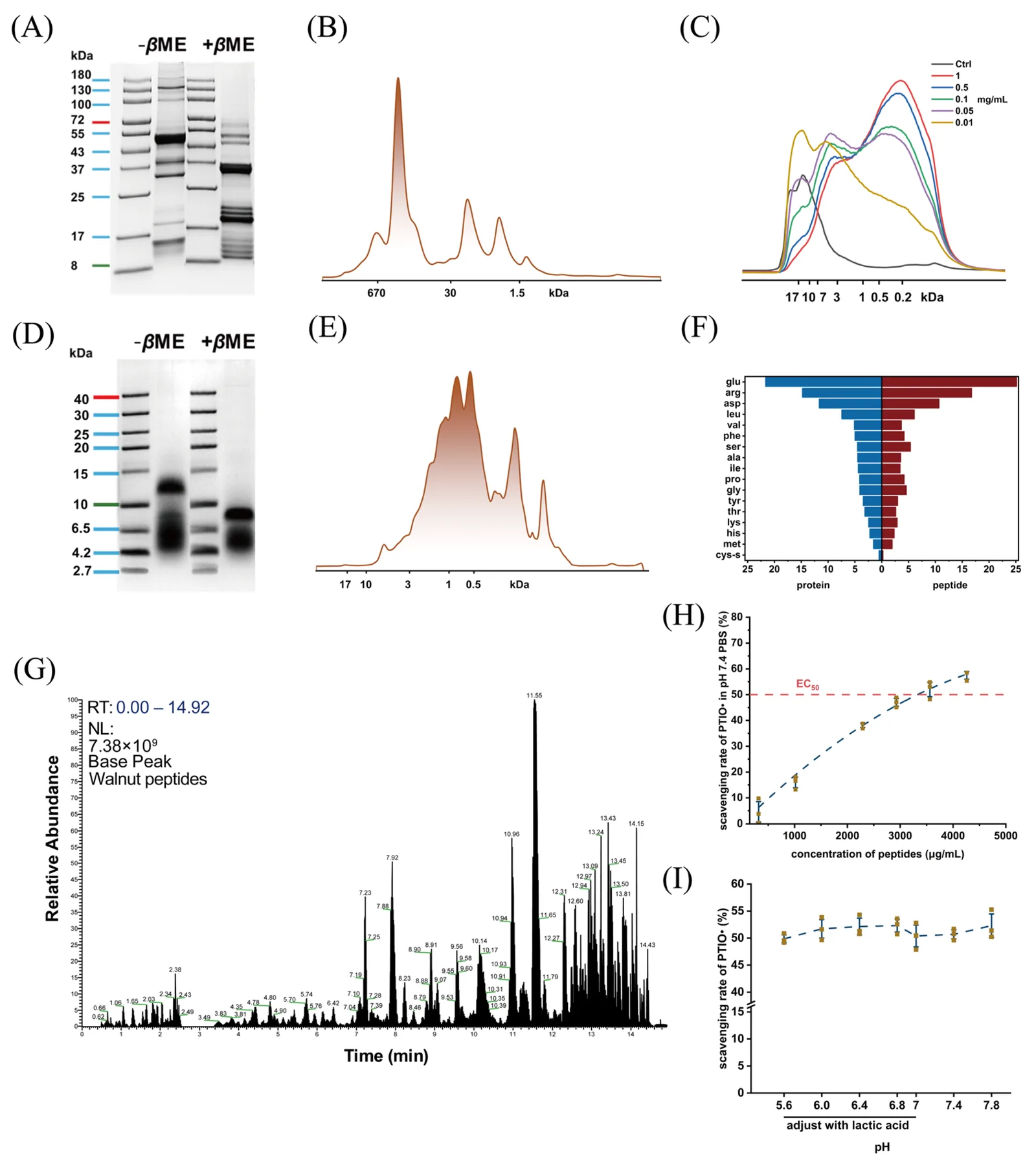
Physicochemical characterization of walnut protein and its enzymatic hydrolysates. (**A**) Tris-Gly SDS-PAGE with 4–20% separating gel, and (**B**) SEC-HPLC using Shodex PROTEIN KW-804 profiles showing the molecular weight distribution of walnut protein. Lanes 1 and 2 represent protein without (−) and with (+) *β*-mercaptoethanol (*β*-ME), respectively. (**C**) Changes in peptide molecular weight after enzymatic hydrolysis with 0 (Ctrl), 0.01, 0.05, 0.1, 0.5, and 1 mg/mL trypsin, respectively, for 5 min. (**D**) Tricine SDS-PAGE and (**E**) SEC-HPLC using TSKgel^®^ G2000_SWXL_ analysis of the molecular weight distribution of peptides after hydrolysis with 0.05 mg/mL trypsin for 5 min followed by 30 kDa ultrafiltration. Lanes 1 and 2 represent peptides without (−) and with (+) β-mercaptoethanol (*β*-ME), respectively. (**F**) Changes in amino acid composition from walnut protein to final peptides. (**G**) Peptide sequencing via LC-MS/MS using an Orbitrap Astral mass spectrometer. (**H**) EC_50_ values obtained from PTIO• radical-scavenging activity. (**I**) Effect of lactic acid on the EC_50_ of the peptides. SEC-HPLC, size-exclusion high-performance liquid chromatography; EC_50_, half-maximal effective concentration; PTIO•, 2-phenyl-4,4,5,5-tetramethylimidazoline-1-oxyl 3-oxide.

Kjeldahl nitrogen determination revealed that the walnut protein prepared in this experiment contained 74% protein, with molecular weights predominantly in the range of 8–55 kDa. Additionally, a high-molecular-weight fraction (>100 kDa) was identified, with subunits ranging from 8 to 37 kDa (Figure 1A). SEC analysis indicated that, when untreated with boiling denaturation or SDS, walnut protein predominantly consisted of medium-to-high-molecular-weight components (>30 kDa), alongside endogenous peptides or degradation products (<1.5 kDa) generated during extraction (Figure 1B). To obtain peptide fractions with a broader molecular weight distribution, we hydrolyzed walnut protein with varying trypsin concentrations for 5 min. As depicted in Figure 1C, the control sample (boiled to remove high-molecular-weight proteins, without trypsin) mainly contained proteins above 7 kDa. With an increasing trypsin concentration, the molecular weight distribution shifted toward lower molecular weights, reaching a plateau at 0.5 mg/mL, with no significant difference observed between 0.5 and 1 mg/mL treatments. Based on these results, we selected 0.05 mg/mL trypsin for subsequent hydrolysis. The resulting hydrolysate was further subjected to ultrafiltration, yielding a sample with a 66% protein content. Tricine SDS-PAGE revealed peptides ranging from 4.2 to 15 kDa, whereas SEC indicated that the majority were smaller than 3 kDa (Figure 1D,E). Amino acid profiling further showed that, among the five most abundant amino acids in walnut protein, the proportions of glutamic acid and arginine increased, while those of aspartic acid, leucine, and valine decreased following hydrolysis (Figure 1F). Sequencing results (Figure 1G) showed that 1564 peptides with an average local confidence (ALC) >90% were identified in the walnut peptide preparation obtained in this study. The radical-scavenging activity of the walnut peptides against PTIO• was evaluated as illustrated in Figure 1H, the peptides exhibited notable scavenging activity. Furthermore, lactic acid did not significantly alter this scavenging activity (Figure 1I).

### 3.2. In Vivo Effect of Peptide Diet in Mice

**Figure 2:**
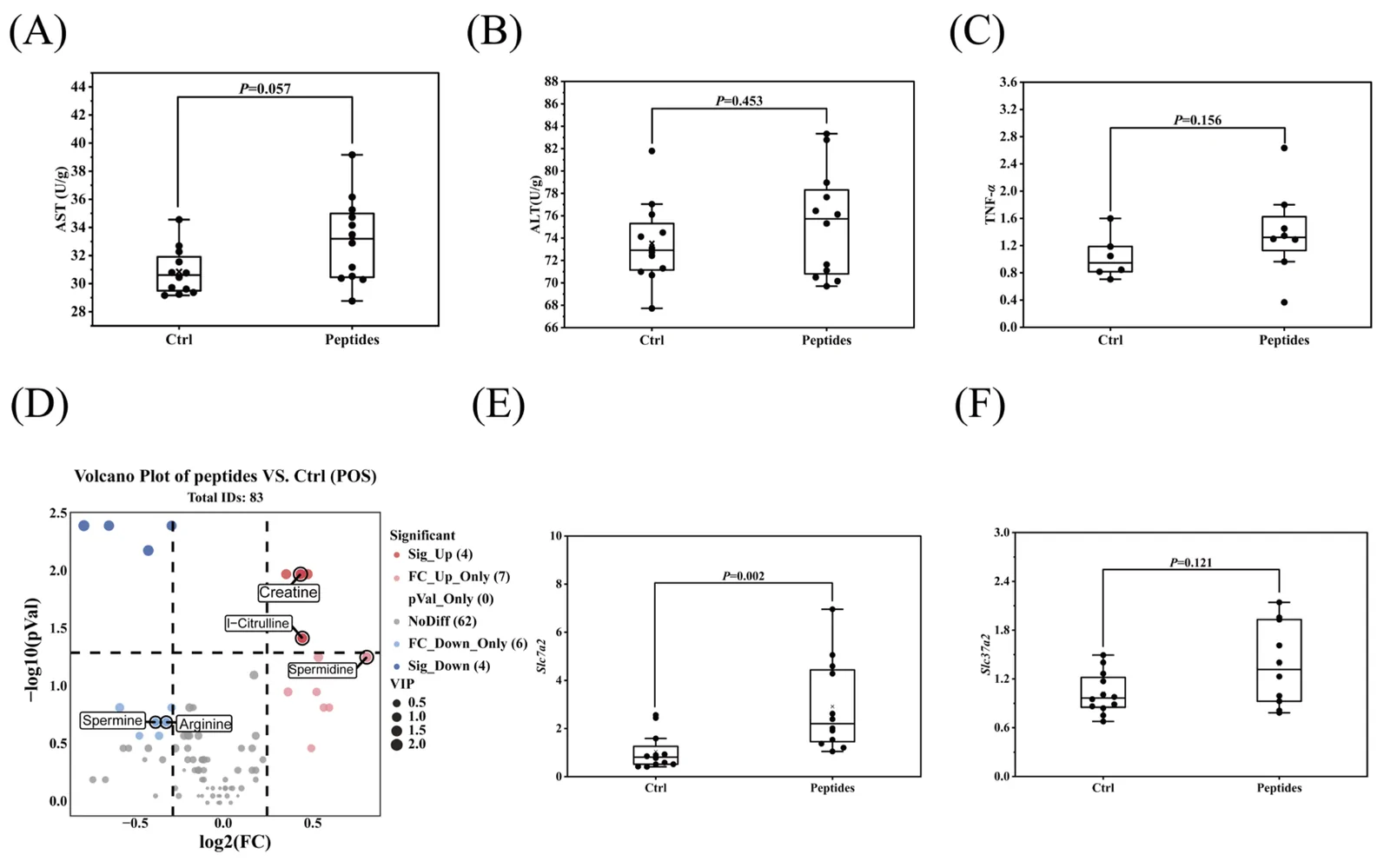
Effects of oral walnut peptide administration on hepatic injury markers, inflammation, metabolites, and transporter gene expression in mice. (**A**) AST, (**B**) ALT, and (**C**) TNF-α mRNA expression in the liver after walnut peptide administration. (**D**) Differential metabolites detected in the positive ion mode. (**E**) Relative mRNA expression of *Slc7a2* and (**F**) *Slc37a2* in the liver. Data are presented as means ± SD (n = 12). All mRNA expression was normalized to the internal reference gene *β*-actin. Statistical comparisons were performed using the Mann–Whitney *U* test.

Compared with the control group, no significant alterations in hepatic aspartate aminotransferase (AST) (Figure 2A) or alanine aminotransferase (ALT) (Figure 2B) levels were observed in mice administered walnut peptides via gastric lavage, indicating that peptide intake did not induce hepatocellular injury. However, hepatic TNF-α expression showed an increasing trend in the peptide-treated group (Figure 2C). Using a previously established broad-targeted metabolomics approach [36], differential metabolites in mouse livers were identified (Figure 2D). Multiple compounds involved in arginine metabolism were found to be altered, including arginine (VIP = 1.26, FC = 0.81, *p* = 0.20), citrulline (VIP = 1.84, FC = 1.38, *p* = 0.04), spermine (VIP = 1.10, FC = 0.78, *p* = 0.20), spermidine (VIP = 1.77, FC = 1.76, *p* = 0.05), and creatine (VIP = 1.98, FC = 1.37, *p* = 0.01). Concurrently, the expression of *Slc7a2*, a gene encoding a transporter involved in arginine uptake, was significantly upregulated (*p* = 0.002) (Figure 2E). In addition, *Slc37a2*, a transporter associated with macrophage inflammation, showed an elevated expression trend in the peptide-treated group compared to the control (Figure 2F).

### 3.3. Effects of Peptides on Macrophage Polarization

**Figure 3:**
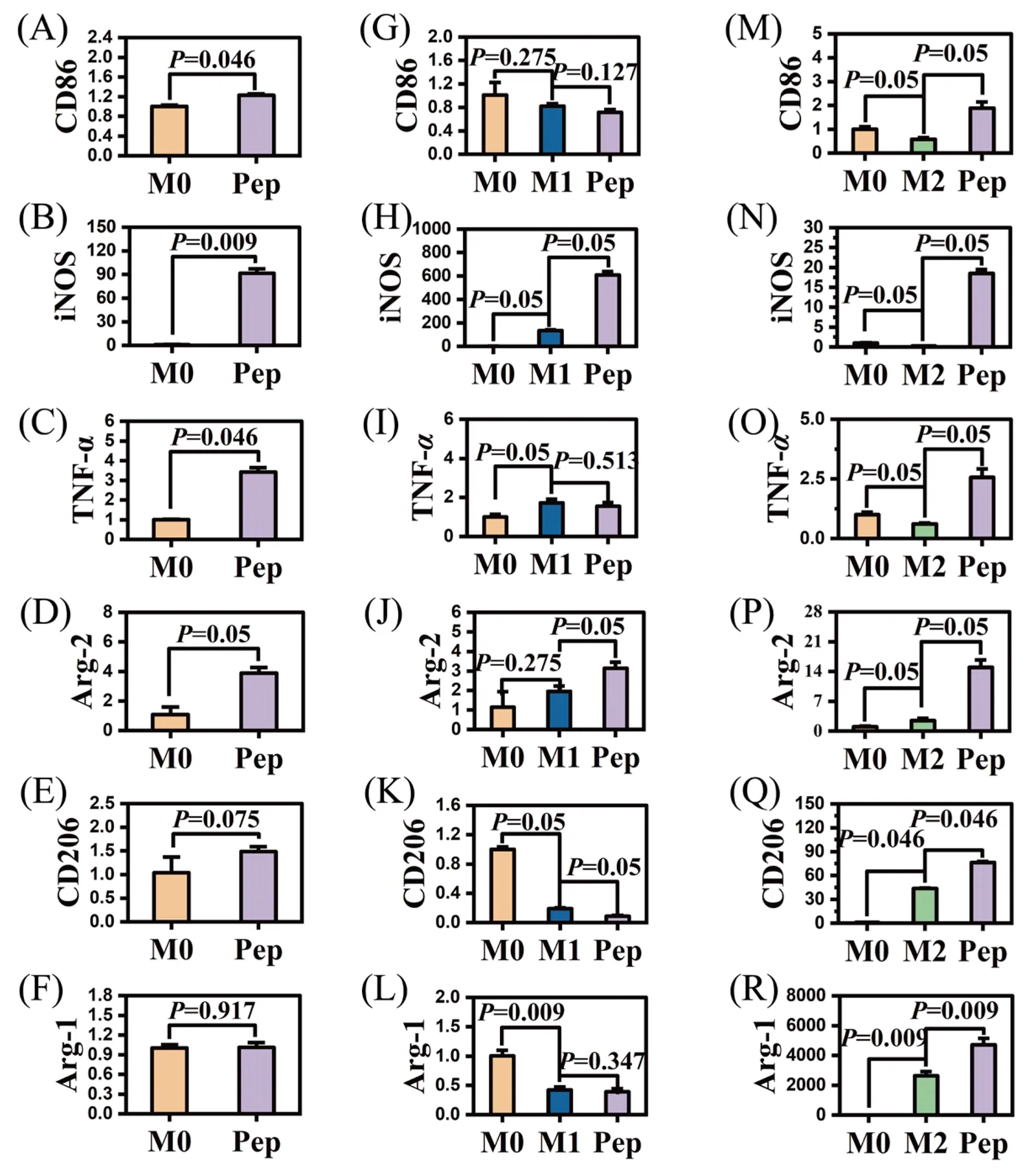
Effects of walnut peptides on the relative mRNA expression of inflammation and arginine-metabolism-related genes in the iBMDM macrophage cell line under different polarization states (n = 3). Murine iBMDM cells were induced into M0 (unstimulated/resting), M1 (pro-inflammatory, stimulated with LPS), and M2 (anti-inflammatory/repair, stimulated with IL-4 + IL13) subtypes to evaluate the modulatory effects of peptide intervention. Panels (**A**–**F**) represent gene expression changes in M0 macrophages; panels (**G**–**L**) represent those in M1 macrophages; and panels (**M**–**R**) represent those in M2 macrophages. All data are normalized to the internal reference gene *β*-actin, expressed as the mean ± SD, and analyzed via a Mann–Whitney *U* test.

In unactivated (M0) iBMDMs, walnut peptide treatment significantly upregulated the M1-associated markers CD86 (Figure 3A), iNOS (Figure 3B), and TNF-α (Figure 3C), while the M2-associated markers CD206 (Figure 3E) and Arg-1 (Figure 3F) remained unchanged, indicating a phenotypic shift toward the M1 phenotype. In LPS-induced M1 macrophages, peptide treatment further elevated iNOS expression (Figure 3H) among M1 genes, with no significant effect on CD86 (Figure 3G) or TNF-α (Figure 3I). Among M2 genes, CD206 was substantially downregulated (Figure 3K), whereas Arg-1 remained unaltered (Figure 3L). These results suggest that walnut peptides promote and deepen the M1-biased phenotype in both uninduced and M1-polarized macrophages. In IL-4/IL-13-induced M2 (immunosuppressive) macrophages, peptide treatment reversed the immunosuppressive phenotype, as reflected by upregulated expression of the M1-associated genes CD86 (Figure 3M), iNOS (Figure 3N), and TNF-α (Figure 3O). However, the M2-associated genes CD206 (Figure 3Q) and Arg-1 (Figure 3R) were also significantly upregulated, presenting a seemingly paradoxical observation. Similar trends were observed in RAW264.7 macrophages (Supplementary Figure S1A–D). Flow cytometry analysis under immunosuppressive conditions revealed that walnut peptides increased CD86 protein expression while suppressing CD206 expression; however, the proportion of CD86^+^/CD206^+^ double-positive macrophages was also significantly elevated, accompanied by increased secretion of both TNF-α and IL-10 (Supplementary Figure S1E–M). Notably, Arg-2 (mitochondrial isoform) was significantly upregulated following peptide stimulation in all three cellular models (Figure 3D,J,P), suggesting the context-dependent modulation of arginine metabolic enzymes.

### 3.4. Effects of Peptides on Tumor Cell Viability and Macrophage-Mediated Tumor Killing

**Figure 4:**
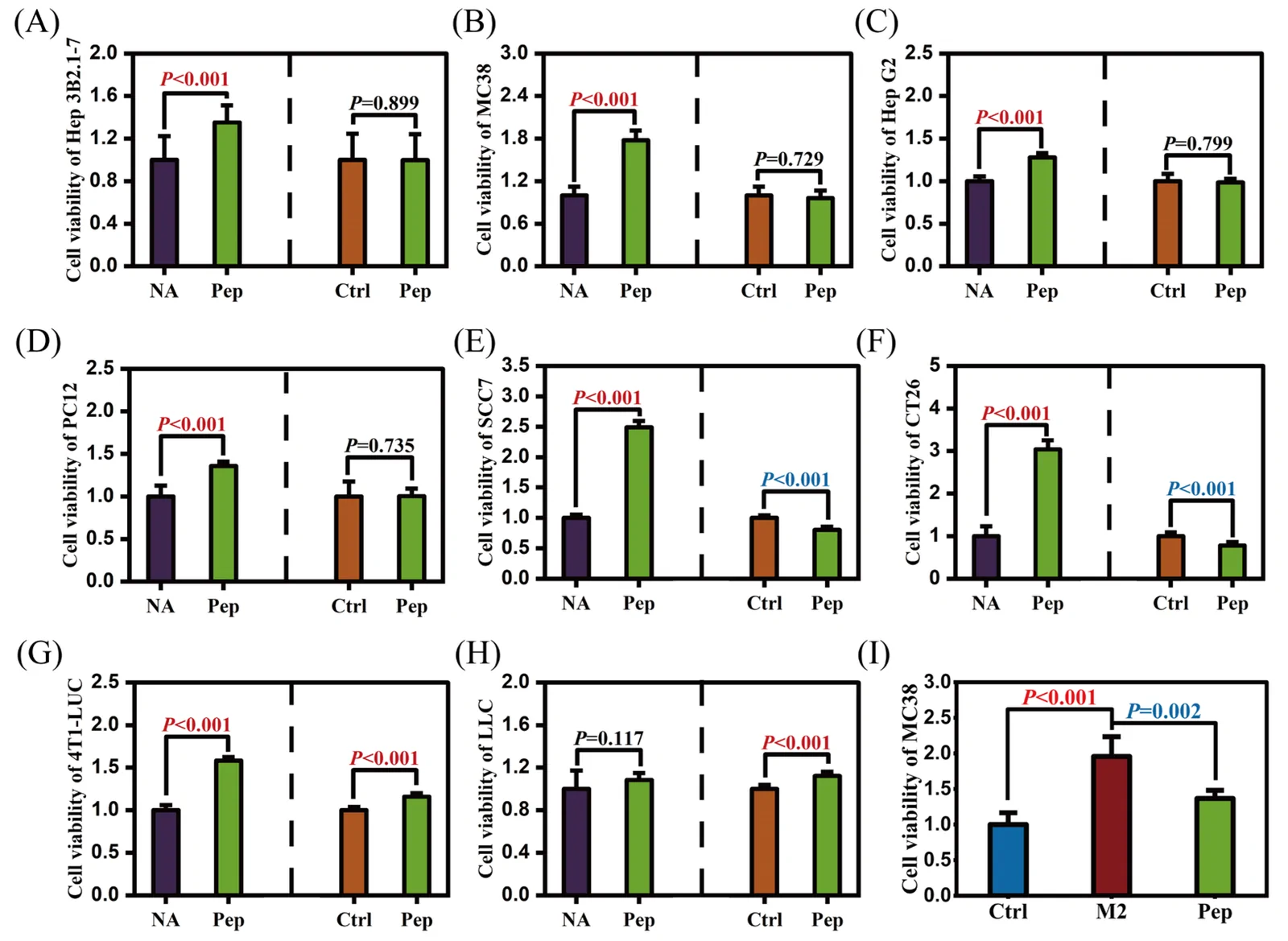
Effects of walnut peptides on cancer cell viability under arginine-modulated conditions and on immune–tumor interactions in a co-culture system. ((**A**–**H**) n = 6) Cell viability of various cancer cell lines after incubation with peptides in arginine-containing medium (right parts of the bar graphs) or arginine-free medium (left parts of the bar graphs). ((**I**) n = 3) Cell viability of MC38 cells in a Transwell co-culture system with immunosuppressed iBMDM cells. NA, control in arginine-free medium; Ctrl, control in arginine-containing medium; Pep, peptides. Statistical comparisons were performed using the Mann–Whitney *U* test.

In the normal culture medium (with sufficient arginine), walnut peptide supplementation did not significantly affect the viability of Hep 3b2.1-7 (Figure 4A), MC38 (Figure 4B), Hep G2 (Figure 4C), or PC12 (Figure 4D) cells. However, walnut peptides inhibited the proliferation of SCC7 (Figure 4E) and CT26 (Figure 4F) cells, while promoting that of 4T1-LUC (Figure 4G) and LLC (Figure 4H) cells. In the arginine-deficient medium, walnut peptides promoted proliferation across all tested tumor cell lines. To assess whether peptide-induced macrophage repolarization translates into functional anti-tumor activity, macrophages were first treated with walnut peptides and subsequently co-cultured with MC38 cells in a Transwell system, which prevents direct peptide–tumor cell contact. As shown in Figure 4I, the tumor-promoting effect of M2 macrophages on MC38 proliferation was effectively reversed following walnut peptide treatment, leading to the significant inhibition of tumor cell growth via macrophage-mediated immune regulation.

### 3.5. Impact of Peptides on Macrophage Untargeted Metabolomics

**Figure 5:**
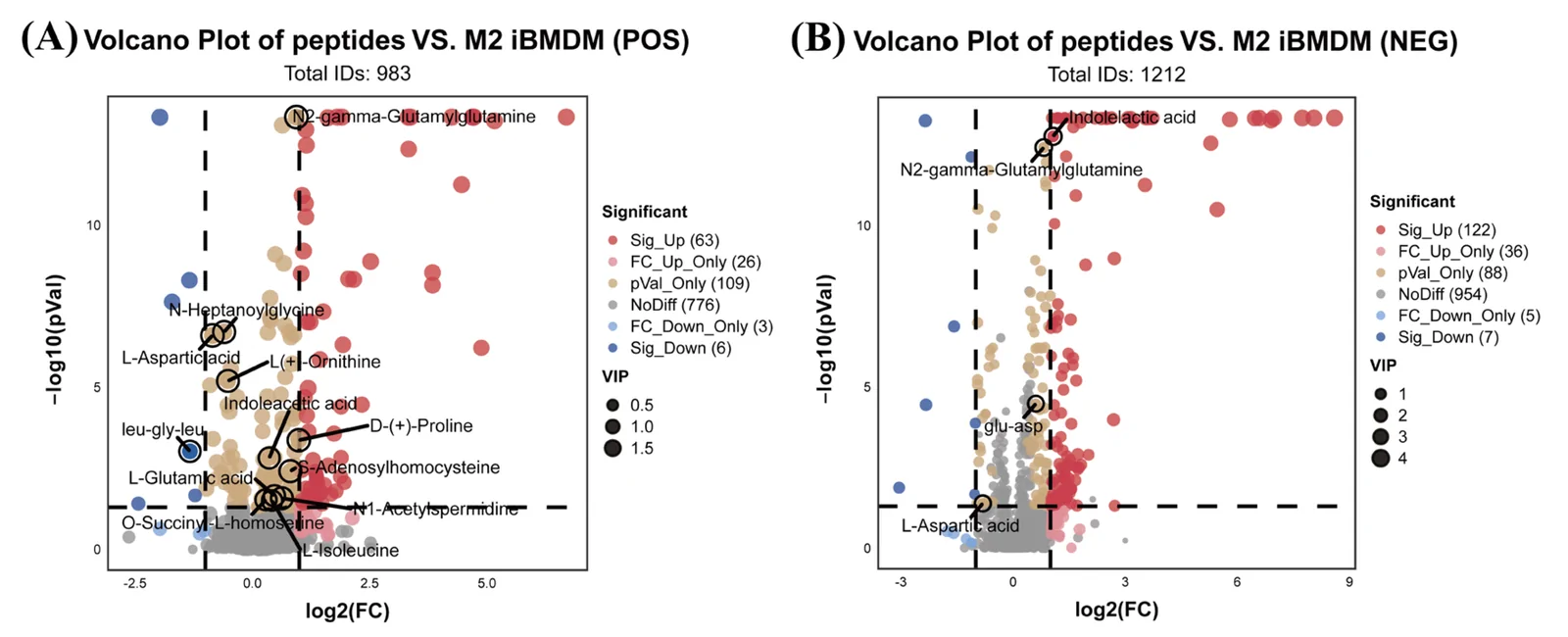
Effects of walnut peptides on the metabolic profile of immunosuppressed iBMDM macrophages (n = 6). (**A**,**B**) Volcano plots showing the distribution of differentially expressed metabolites between the peptide-treated group and the immunosuppressed model group, detected in positive and negative ion modes, respectively.

Untargeted metabolomics analysis was performed on iBMDMs to obtain a comprehensive metabolic profile. In the positive ion mode (Figure 5A), multiple metabolites involved in amino acid metabolism were significantly altered. Among those directly linked to arginine metabolism were L-ornithine (Log_2_FC = −0.52, *p* = 6.3 × 10^−6^, VIP = 1.71) and S-adenosylhomocysteine (Log_2_FC = 0.81, *p* = 0.0039, VIP = 1.72). Metabolites associated with the glutamine–glutamate–aspartate axis were also affected, including L-glutamic acid (Log_2_FC = 0.47, *p* = 0.022, VIP = 1.52), L-aspartic acid (Log_2_FC = −0.85, *p* = 2.5 × 10^−7^, VIP = 1.89), and N2-γ-glutamylglutamine (Log_2_FC = 0.94, *p* = 4.6 × 10^−14^, VIP = 1.89). Additionally, metabolites implicated in proline metabolism, such as L-proline (Log_2_FC = −0.84, *p* = 0.00039, VIP = 1.62), and tryptophan-derived indoleacetic acid (Log_2_FC = 0.36, *p* = 0.0015, VIP = 1.52), were significantly altered. In the negative ion mode (Figure 5B), the downregulation of L-aspartic acid (Log_2_FC = −0.81, *p* = 0.041, VIP = 1.22) and upregulation of N2-α-glutamylglutamine (Log_2_FC = 0.82, *p* = 3.8 × 10^−13^, VIP = 1.42) and Glu-Asp (Log_2_FC = 0.61, *p* = 3.3 × 10^−5^, VIP = 1.12) were observed, corroborating the positive ion data.

### 3.6. Impact of Peptides on Macrophage Arginine Metabolism

**Figure 6:**
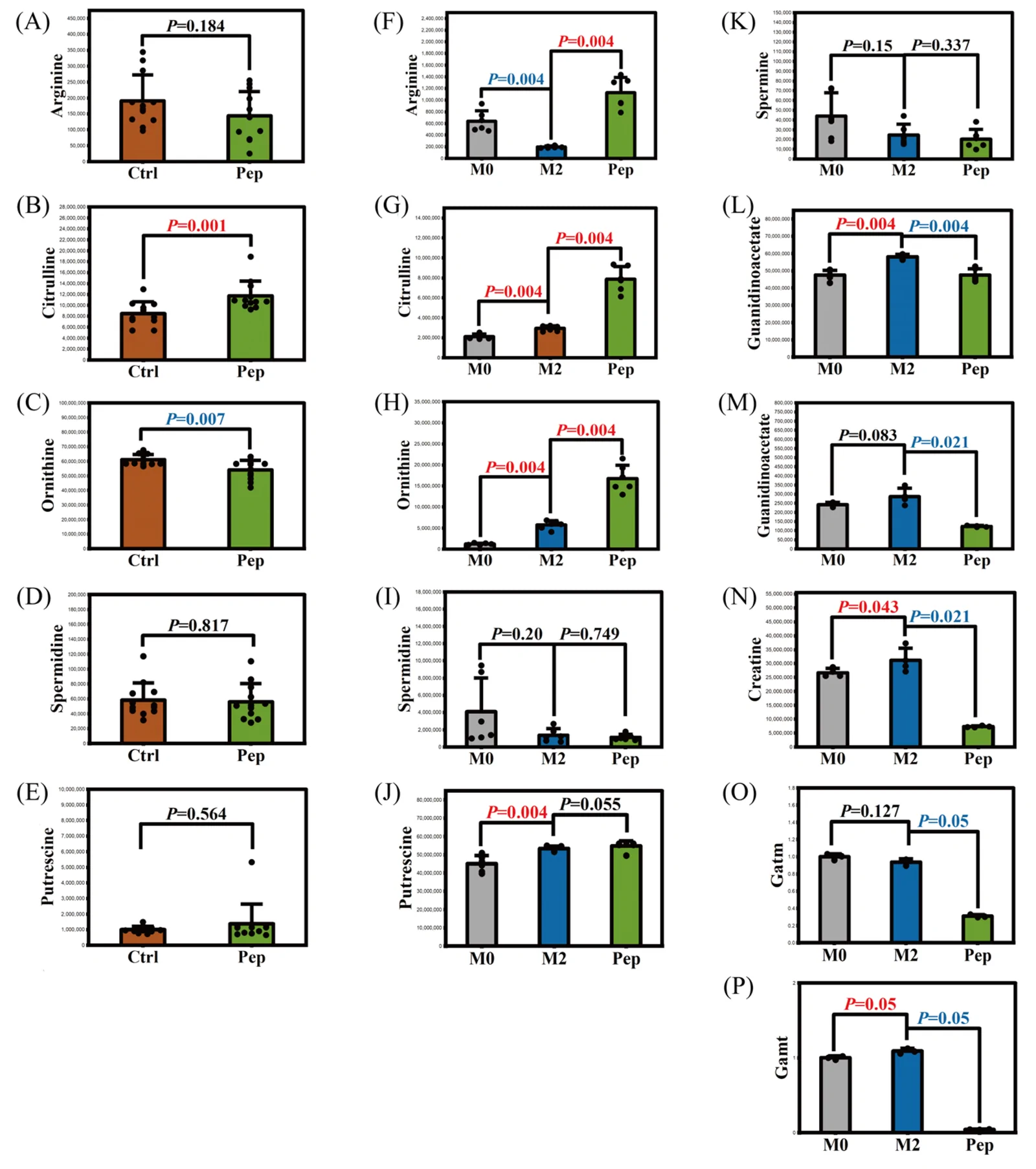
Effects of walnut peptides on arginine-related metabolites and metabolic gene expression in vivo and in vitro. ((**A**–**E**) n = 12) Levels of arginine and related metabolites in mouse liver homogenates. ((**F**–**L**) n = 6) and ((**M**,**N**) n = 4) Levels of the corresponding metabolites in iBMDM and RAW264.7 cell lysates, respectively. Relative mRNA expression of Gatm ((**O**) n = 3) and Gamt ((**P**) n = 3) in RAW264.7 cells. Ctrl, control; Pep, peptides. Statistical comparisons were performed using the Mann–Whitney *U* test.

To validate the metabolomic findings, targeted metabolomics was employed to quantify arginine and its key metabolites in mouse liver tissue and iBMDM cells. In mouse liver tissue, arginine levels exhibited a decreasing trend (Figure 6A), whereas citrulline levels were significantly elevated (Figure 6B), consistent with the pseudo-targeted results (Figure 2). Ornithine levels (Figure 6C) were significantly reduced, while spermidine (Figure 6D) and putrescine (Figure 6E) showed no notable changes. Upon IL-4/IL-13-induced polarization of iBMDMs into M2 macrophages, intracellular arginine decreased (Figure 6F), whereas citrulline (Figure 6G), ornithine (Figure 6H), putrescine (Figure 6J), and guanidinoacetic acid (GAA) (Figure 6L) were significantly increased; spermidine (Figure 6I) and spermine (Figure 6K) remained unaltered. Following walnut peptide intervention, intracellular arginine rose, and the accumulation of citrulline and ornithine increased markedly above the M2 baseline, while GAA levels were substantially downregulated. Putrescine, spermine, and spermidine showed no discernible changes. Consistent results were obtained in RAW264.7 macrophages: walnut peptides inhibited the intracellular accumulation of GAA (Figure 6M) and creatine (Figure 6N), an effect associated with the suppression of GAA (Figure 6O) and creatine (Figure 6P) synthesis enzymes.

### 3.7. Effects of Peptides on the Transcriptome of Macrophages

**Figure 7:**
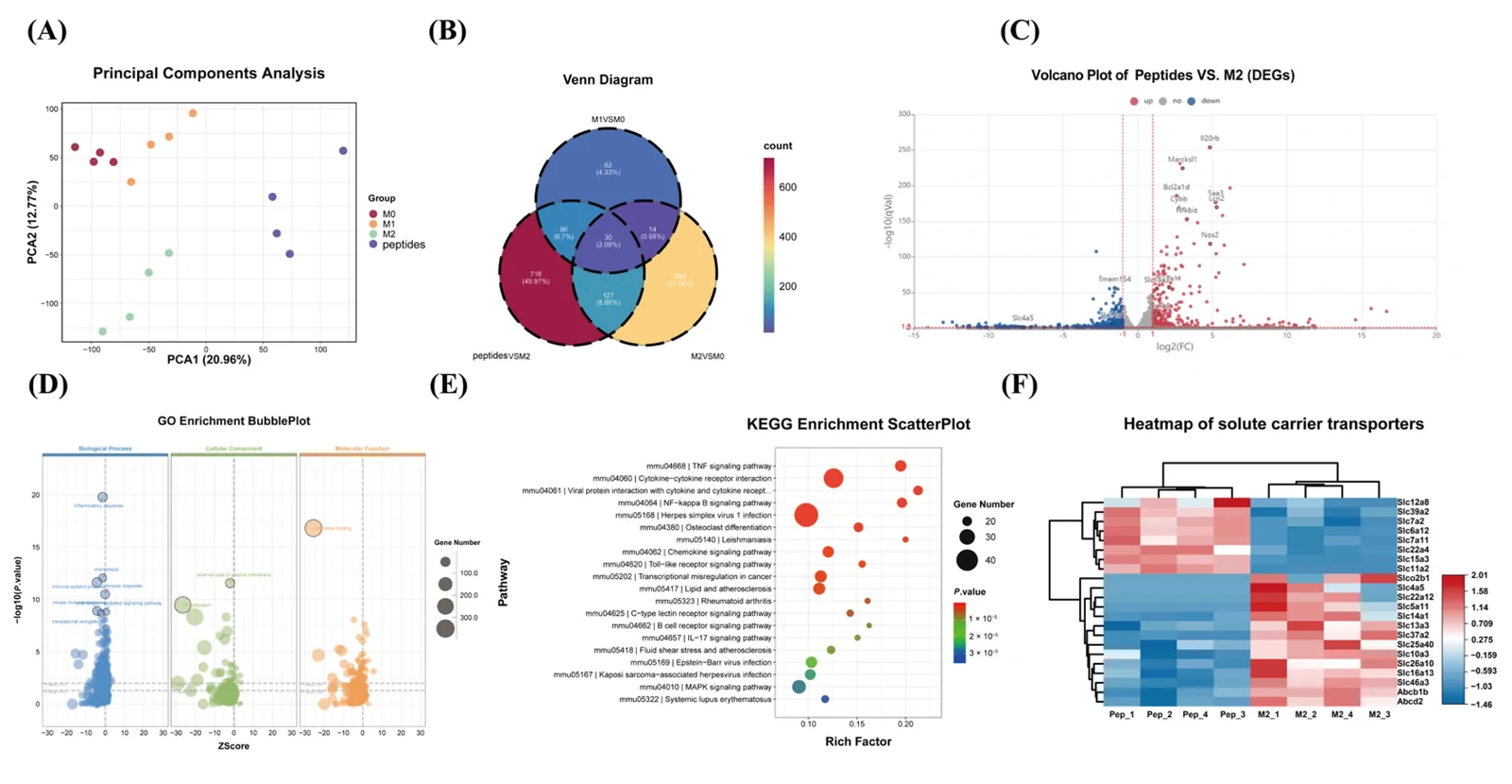
Transcriptomic profiling of macrophages under different polarization states and after walnut peptide treatment. (**A**) Principal component analysis (PCA) of the transcriptomes of M0, M1, M2, and peptide-treated M2 (peptides) groups (n = 4 per group). (**B**) Venn diagram showing the overlap of differentially expressed genes (DEGs) among the M2 vs. M0, M1 vs. M0, and peptides vs. M2. (**C**) Volcano plot of DEGs between the peptides and M2 groups. (**D**) GO enrichment analysis of DEGs between the peptides and M2 groups. (**E**) KEGG pathway enrichment analysis of DEGs between the peptides and M2 groups. (**F**) Heatmap of solute carrier transporter genes in the peptides and M2 groups.

To complement the metabolomic findings and gain deeper insight into the transcriptional basis of peptide-induced macrophage repolarization, transcriptomic analysis was performed on M0, M1, M2, and peptide-treated M2 macrophages. PCA revealed clear separation of IL-4/IL-13-induced M2 macrophages from M0 and M1 cells (Figure 7A), confirming substantial differences in their gene expression profiles (Figure 7B). Peptide-treated M2 macrophages formed a distinct cluster separate from both the M2 model group and LPS-induced M1 macrophages, indicating that walnut peptides act on M2 macrophages via a mechanism distinct from that of LPS. Among the DEGs between LPS-induced M1 and M0 macrophages, only 8% overlapped with those between peptide-treated M2 and M0 macrophages, while approximately 11% of the DEGs between M2 and M0 overlapped with those in peptide-treated M2 cells. Following peptide treatment, 337 genes were upregulated and 632 were downregulated (Figure 7C). GO enrichment analysis (Figure 7D) revealed that the top five biological processes included signal transduction (GO:0007165), the regulation of transcription (GO:0006355, GO:0045944, GO:0006357), and immune system processes (GO:0002376, GO:0006954, GO:0006955). Notably, cellular component analysis identified membrane-associated terms (GO:0016020, GO:0005886) among the top categories. KEGG pathway analysis (Figure 7E) showed that the cytokine–cytokine receptor interaction (mmu04060), NF-kappa B signaling (mmu04064), and TNF signaling (mmu04668) were among the most significantly enriched pathways. Transporter-related genes were notably affected, and *Slc7a2*, an arginine transporter, was upregulated in peptide-treated macrophages—consistent with the increased hepatic *Slc7a2* expression observed in peptide-fed mice (Figure 2F).

## 4. Discussion

In this study, we systematically characterized walnut protein and its tryptic hydrolysates, laying a physicochemical foundation for subsequent functional investigations. The molecular weight distribution of the intact protein (8–55 kDa, plus a >100 kDa fraction) was consistent with previous reports [38], confirming the reliability of our extraction protocol. A moderate trypsin concentration (0.05 mg/mL) was deliberately chosen to generate hydrolysates with a broad molecular weight distribution, aiming to preserve diverse immunomodulatory epitopes rather than complete conversion to oligopeptides, which is commonly employed in cost-effective protein processing [25]. Although Tricine-SDS-PAGE and SEC showed discrepancies in molecular weight estimation of the ultrafiltered fraction (4.2–15 kDa vs. <3 kDa), this was mainly attributed to the staining limitations and resolution constraints of PAGE, which do not compromise functional assessments. Further mass spectrometric sequencing results showed that the molecular weight distribution of the peptides ranged from 382 to 3730 Da. Hydrolysis increased the proportions of glutamic acid and arginine; notably, arginine is a key substrate for metabolic reprogramming in macrophages, providing an early clue for our mechanistic focus. Importantly, walnut peptides retained radical-scavenging activity in the presence of lactic acid and exhibited superior stability compared to peptides from other sources, indicating resistance to lactic-acid-induced destabilization [39]. Given the lactic-acid-rich tumor microenvironment, this property positions walnut peptides as promising candidates for reversing the immunosuppressive phenotypes of tumor-associated macrophages and provided the material basis for all subsequent in vivo and in vitro experiments.

Given that walnut peptides were administered orally, their in vivo effects involve integrated physiological processes such as absorption, tissue distribution, and the hepatic immune microenvironment. Therefore, an animal study was performed first to evaluate the systemic effects of walnut peptides. The oral administration of walnut peptides did not elevate hepatic AST/ALT levels, demonstrating a favorable safety profile in vivo. Although AST exhibited a slight upward trend, this was accompanied by increased TNF-α expression; given the immunosuppressive nature of the hepatic microenvironment [40,41,42], such inflammatory elevation may be beneficial for reversing immunosuppression and controlling tumor progression [43,44]. Pseudo-targeted metabolomics revealed that multiple arginine-related metabolites (arginine, citrulline, spermine, spermidine, and creatine) were altered in the liver, and hepatic *Slc7a2* expression was significantly upregulated, suggesting that walnut peptides systemically modulate arginine metabolism. However, since hepatocytes constitute approximately 70% of liver cells and also express *Slc7a2*, these metabolic changes cannot be unequivocally attributed to immune cells. Notably, macrophages represent over 80% of hepatic immune cells [41], and *Slc37a2*—a transporter primarily expressed in macrophages [45]—was upregulated in the peptide-treated group, further supporting an impact of walnut peptides on hepatic macrophages. These in vivo findings directly guided our subsequent in vitro investigations on macrophage function.

We then examined the direct effects of walnut peptides on macrophages at distinct polarization states. In M0 macrophages, peptide treatment significantly upregulated M1 markers (CD86, iNOS, TNF-α) without affecting M2 markers, indicating a pro-inflammatory shift. In LPS-induced M1 macrophages, the peptide further deepened the M1 phenotype (elevated iNOS, reduced CD206). In IL-4/IL-13-induced M2 macrophages, walnut peptides not only elevated M1 markers but also concurrently upregulated certain M2 markers (CD206 and Arg-1)—a seemingly paradoxical observation that was corroborated by the presence of CD86^+^/CD206^+^ double-positive macrophages in flow cytometry [46]. We propose three non-mutually exclusive mechanisms: (i) negative feedback regulation upon reaching an inflammatory threshold [47], (ii) differential uptake of peptide fractions by distinct macrophage subpopulations [48], and (iii) simultaneous activation of both M1 and M2 pathways within the same cell. Notably, most food-derived immunomodulators have been evaluated using M0 or LPS-induced models [49,50], with few studies addressing IL-4/IL-13-induced M2 macrophages. Our findings demonstrate that immunostimulatory activity identified in M0 macrophages cannot be directly extrapolated to the modulation of immunosuppressive macrophages in the tumor microenvironment. Moreover, walnut peptides directly influenced tumor cell behavior in a cell-type- and microenvironment-dependent manner: they inhibited SCC7 and CT26 but promoted 4T1-LUC and LLC proliferation, and under arginine-deficient conditions, they promoted all tested lines—underscoring a potential risk of heterogeneous peptide mixtures and reinforcing the need for the targeted purification of immunostimulatory core sequences. Critically, using a Transwell co-culture system that eliminated direct peptide–tumor contact, we demonstrated that walnut peptide-treated M2 macrophages effectively reversed their tumor-promoting propensity and inhibited MC38 growth. This immune-centric strategy circumvents the limitations of direct cytotoxic approaches [16,23,24] and represents a significant advancement, confirming, at both the transcriptional and functional levels, that our peptide preparation contains core sequences capable of reprogramming immunosuppressive macrophages toward an inflammatory phenotype.

To elucidate the underlying mechanisms, we performed untargeted and targeted metabolomics, together with transcriptomics. Untargeted metabolomics revealed that walnut peptide treatment broadly reprograms amino acid metabolism in macrophages, with multiple altered metabolites (L-proline, L-glutamic acid, L-aspartic acid, L-ornithine, indoleacetic acid) previously linked to macrophage polarization [42,51,52,53,54,55]. A notable apparent paradox emerged: L-ornithine was decreased in metabolomic data despite the upregulation of Arg-1 expression, which may reflect increased metabolic flux from ornithine toward downstream pathways (e.g., polyamine and proline biosynthesis) [56] rather than reduced arginase activity. Targeted metabolomics precisely quantified these changes and re-confirmed that peptide treatment elevated intracellular arginine, citrulline, and ornithine above the M2 baseline, while substantially downregulating GAA and creatine, associated with suppressed expression of their synthesis enzymes. Citrulline accumulation provides metabolic evidence for repolarization toward a pro-inflammatory phenotype, as citrulline is a co-product of iNOS-mediated arginine oxidation and can be recycled via the citrulline–NO cycle to sustain NO production [57]. The lack of change in putrescine, spermidine, and spermine, despite ornithine accumulation, indicates that peptide treatment did not promote ornithine conversion to polyamines via ODC, partially accounting for ornithine accumulation [58]. The most striking finding was the significant downregulation of GAA and creatine, which is functionally relevant because creatine has been shown to suppress macrophage transition toward an inflammatory phenotype [59]. Thus, the peptide-mediated inhibition of creatine synthesis likely contributes to the observed repolarization. Transcriptomic data further demonstrated that walnut peptides induce distinct transcriptional reprogramming in M2 macrophages, rather than simply mimicking LPS-driven M1 polarization, as evidenced by limited DEG overlap and clear PCA separation. The enrichment of immune-related pathways (NF-κB and TNF signaling) and membrane-associated GO terms, together with upregulation of the arginine transporter *Slc7a2*—consistent with in vivo hepatic findings—supports that enhanced arginine uptake is a key feature. While *Slc7a2* is induced during both M1 and M2 activation [60], our integrated data suggest that arginine imported via upregulated *Slc7a2* is preferentially channeled into pathways beyond iNOS and Arg-1 metabolism, specifically pointing to the arginine-to-creatine conversion axis as the central metabolic node. Taken together, our multi-omics and functional data suggest that walnut peptides limit the arginine→GAA→creatine pathway, thereby redirecting arginine metabolism toward a pro-inflammatory profile. This mechanistic insight, obtained without prior target bias, provides a valuable framework for identifying bioactive components that may have been overlooked in earlier studies using conventional fractionation or disease-target-guided purification. We interpret these pathway enrichments with caution, as the peptide preparation is a complex mixture containing thousands of segments with potential synergistic or antagonistic activities, consistent with reports that different fractions from the same hydrolysate can exhibit opposing effects [11,12]. Therefore, definitive pathway assignment and sequence identification will require future validation using sequence-defined peptides. Nevertheless, our current data clearly establish the arginine–GAA–creatine metabolic axis as a novel target for food-derived peptide-mediated macrophage repolarization, distinct from the conventional iNOS/Arg-1 dichotomy.

## 5. Conclusions

This study was undertaken with two primary objectives: first, to determine whether walnut proteolytic hydrolysate harbors core peptide sequences capable of reversing the immunosuppressive phenotype of macrophages; and second, to preliminarily investigate the underlying mechanisms. Our findings demonstrate that walnut peptides promote the polarization of uncommitted (M0) macrophages toward an inflammatory (M1-like) phenotype, further elevate M1-associated gene expression in LPS-induced macrophages, and effectively reverse the IL-4/IL-13-induced immunosuppressive state, thereby restoring the anti-tumor functionality of macrophages. Both in vivo and in vitro evidence indicate that walnut peptides modulate the arginine metabolic pathway. In addition to influencing the canonical arginine–citrulline and arginine–ornithine axes, walnut peptides also exert an impact on the arginine-to-creatine conversion pathway, specifically by limiting the synthesis of GAA and creatine. We therefore propose that modulation of the arginine-to-creatine conversion pathway may serve as a metabolic readout for the future isolation and purification of peptide sequences responsible for reversing immunosuppression. Despite these encouraging findings, several key questions remain to be addressed. Once sequence-defined peptides are identified through screening based on intervention in the arginine-to-creatine metabolic axis, their oral pharmacokinetic profiles should be carefully characterized, and their differential uptake between macrophages and tumor cells should be assessed to determine whether additional formulation and modification strategies—such as encapsulation or structural modification—are required to ensure adequate oral bioavailability, stability, and targeting of the active peptides.

## Data Availability

The original contributions presented in this study are included in the article/Supplementary Materials. Further inquiries can be directed at the corresponding author.

## Supplementary Materials

The following supporting information can be downloaded at: https://www.mdpi.com/article/10.3390/foods15214014/s1, Figure S1: After intervention with walnut peptides, relative expression of inflammation-related genes (**A**–**D**), proteins (**E**–**K**), and cytokines (**L**–**M**) in the RAW264.7 cell line. (**E**–**H**) The gating strategy for macrophages of M0, M1, M2, and walnut peptide-intervened M2 phenotypes in flow cytometric analysis; Table S1: Mouse primers synthesized by Sangon Biotech (Shanghai) Co., Ltd., used for qPCR analysis.

## Abbreviations

The following abbreviations are used in this manuscript:

GAA	guanidinoacetic acid
CTX	cyclophosphamide
TME	tumor microenvironment
iBMDM	immortalized bone marrow-derived macrophage
