# Supplementary material for: Metabolic Reprogramming of Macrophages by Walnut Peptides: Interference with Arginine-to-Creatine Conversion
Source: Foods. 2026 Nov 9;15(21):4014. doi: 10.3390/foods15214014 (PMC13762997; doi:10.3390/foods15214014)
Supplement: Supplementary file 1 [file foods-15-04014-s001.zip › foods-4612365-supplementary.pdf]

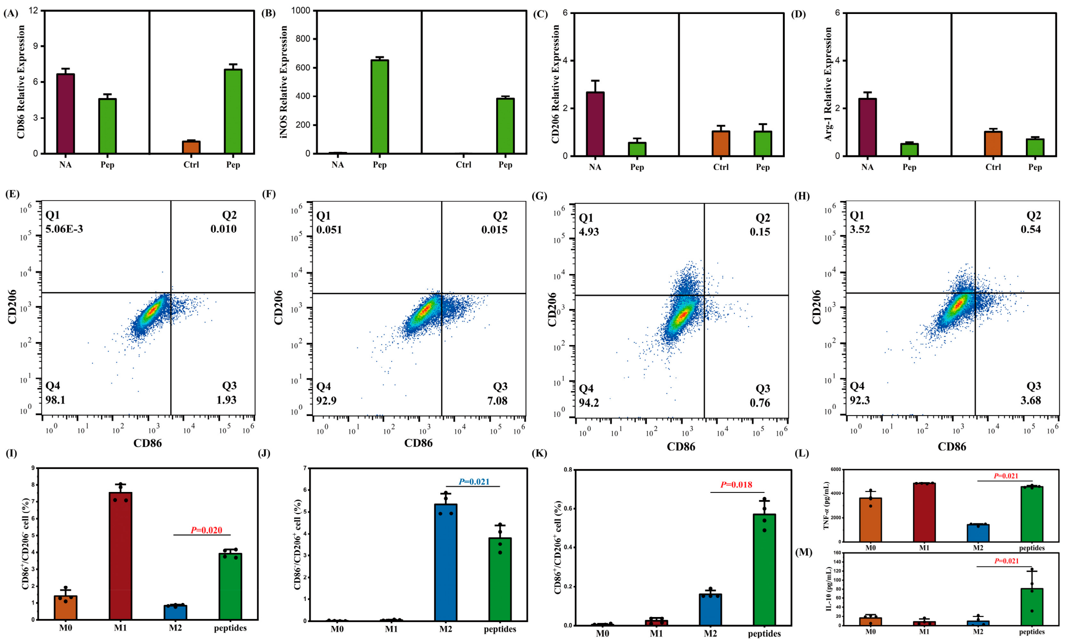

Supplementary Figure S1. After intervention with walnut peptides， relative expression of inflammation-related genes (A-D), protein (E-K) and cytokines (L-M) in RAW264.7 cell line. E–H, the gating strategy for macrophages of M0, M1, M2, and walnut pep-tides-intervened M2 phenotypes in flow cytometric analysis.

Table S1. Mouse primers synthesized by Sangon Biotech (Shanghai) Co., Ltd. used for qPCR analysis.

Genes	primer
CD206 F	AGACGAAATCCCTGCTACTG
CD206 R	CACCCATTCGAAGGCATTC
CD86 F	ACGGAGTCAATGAAGATTTCCT
CD86 R	GATTCGGCTTCTTGTGACATAC
iNOS F	GTTCTCAGCCCAACAATACAAGA
iNOS R	GTGGACGGGTCGATGTCAC
Arg1 F	TTGGGTGGATGCTCACACTG
Arg1 R	GTACACGATGTCTTTGGCAGA
Arg2 F	GGATCCAGAAGGTGATGGAA
Arg2 R	AGAGCTGACAGCAACCCTGT
Slc7a2 F	GTGTGCCTTGTATTACTCCTGGC
Slc7a2 R	CCACCATGACAAAGAGAAGGACC
β-actinF	CGTTGACATCCGTAAAGACC
β-actinR	AACAGTCCGCCTAGAAGCAC
TNF-α F	CTGAACTTCGGGGTGATCGG
TNF-α R	GGCTTGTCACTCGAATTTTGAGA
Gatm F	TGGTGCCAAGTGGACAACAGCA
Gatm R	GCAAGGCTCAAACTCAGTCGTC
Gamt F	TGCCTGACGGTCACTTTGATGG
Gamt R	GACGTGAGGTTGCAGTAGGTGA
